# Prognostic Value of Urinary N-Acetyl-β-d-Glucosaminidase as a Marker of Tubular Damage in Patients with Heart Failure and Mitral Regurgitation

**DOI:** 10.31083/j.rcm2408219

**Published:** 2023-07-31

**Authors:** Tingting Zhao, Guanzhong Chen, Shiyu Zhu, Chengchen Zhao, Chunna Jin, Yao Xie, Meixiang Xiang

**Affiliations:** ^1^Department of Cardiology, The Second Affiliated Hospital, Zhejiang University School of Medicine, 310009 Hangzhou, Zhejiang, China

**Keywords:** N-acetyl-β-d-glucosaminidase, renal tubular dysfunction, mitral regurgitation, heart failure, cardiorenal syndrome

## Abstract

**Background:**

Mitral regurgitation (MR) has a high prevalence and aggravates hypoperfusion and hypoxia in heart failure (HF). Renal tubular epithelial cells are sensitive to hypoxia, and therefore tubulointerstitial damage is quite common in HF. However, the correlation between tubular dysfunction and MR has not been studied. The aim of this work was to evaluate the prognostic significance of urinary N-acetyl-β-d-glucosaminidase (uNAG), a biomarker of renal tubular damage, in patients with HF and MR.

**Methods:**

This was a prospective cohort study of 390 patients (mean age 64 years; 65.6% male) with uNAG measurement on admission (expressed as urinary NAG/urinary creatinine) and at least 1 year of follow-up data. The pre-defined primary endpoint was the composite of all-cause mortality or rehospitalization for HF after discharge. Cox regression analysis, restricted cubic splines, and subgroup analysis were used to investigate the prognostic value of uNAG modeled as a categorical (quartiles) or continuous (per SD increase) variable.

**Results:**

A total of 153 (39.23%) patients reached the composite endpoint over a median follow-up time of 1.2 years. The uNAG level correlated with the severity of HF and with the incidence of adverse events. In a multivariable Cox regression model, each SD (13.80 U/g⋅Cr) of increased uNAG was associated with a 17% higher risk of death or HF rehospitalization (95% confidence interval, 2–33%, *p* = 0.022), and a 19% higher risk of HF rehospitalization (*p* = 0.027). Subgroup analysis revealed the associations between uNAG and poor prognosis were only significant in younger patients (≤65 years) and in patients without obvious cardiovascular comorbidities.

**Conclusions:**

uNAG levels at admission were associated with the risk of adverse outcomes in patients with HF and MR. Additional studies are needed to further investigate the heart-kidney interaction.

## 1. Introduction

Despite major advances in pharmacotherapies and device treatments, the prognosis for heart failure (HF) remains poor. Persistent left ventricular remodeling and mitral annular dilation cause mitral regurgitation (MR). Secondary/functional mitral regurgitation (FMR) reportedly has a prevalence ranging from 17% to 53% in both acute and chronic HF [1, 2, 3], leading to reduced quality of life, a high mortality rate, and dismal prognosis [4]. Previous studies have suggested that MR may be an indicator of the severity of potential ventricular disease, as well as exerting an effect on disease progression [2].

MR increases the left ventricular preload and decreases the forward flow, resulting in hypofusion and hypoxic damage to renal parenchyma and interstitium. It causes elevated pressures in the left atrial (LA), as well as pulmonary vascular resistance and right-sided heart. These effects transmit to the kidney and lead to increased renal venous and interstitial pressures, thereby contributing to “congestive renal failure” [5]. Activation of the sympathetic nervous system and the renin-angiotensin-aldosterone system due to circulation congestion and volume overload also causes undesirable effects to the kidney. Earlier reports have linked endothelial dysfunction to MR [6] and its potential effects on end organs like the kidney [7].

Several investigators have identified important biomarkers and prognostic factors for HF and MR, including natriuretic peptides, troponin T, the New York Heart Association functional class, anemia, left ventricular ejection fraction <40% and no therapy with renin-angiotensin system inhibitors [8, 9]. Advances in technology have resulted in proteomic-based biomarkers and microRNAs being proposed for MR risk prediction [10]. Baseline renal dysfunction is a common complication and an adverse prognostic factor in patients with HF and severe MR. In turn, HF and MR accelerate the progression to end-stage renal disease (ESRD), thus worsening the prognosis. In contrast, the reduction in regurgitation after transcatheter mitral valve (MV) repair has been associated with improved renal function [11, 12].

N-acetyl-β-d-glucosaminidase (NAG) is a large lysosomal enzyme (130–140 kDa) located mostly in proximal tubules with little filtration from the glomerular basal membrane [13, 14]. Urinary NAG (uNAG) is universally recognized as a reliable biomarker of tubular damage [14] and is known to have important prognostic value for adverse outcomes in multiple conditions including hypertension [15], carotid artery atherosclerosis [16], peripheral arterial disease, diabetes mellitus [17], and various kidney diseases [18]. It has also been reported that the NAG level correlates with the severity and prognosis of HF in patients with acute or chronic HF [19, 20, 21]. So far, however, there are no reports on renal tubular dysfunction and NAG in patients with HF and MR. The aim of the present study was therefore to evaluate the uNAG level as a predictor of adverse events in patients with HF and MR. The results should help to develop new hierarchical metrics and therapeutic targets.

## 2. Materials and Methods

### 2.1 Study Design

This was an observational prospective study with consecutive enrollment at a single-center. Adult HF patients admitted to the Department of Cardiology, the Second Affiliated Hospital, Zhejiang University School of Medicine between July 31, 2019 and November 11, 2021 were included in the study. All patients in which echocardiography suggested the presence of MR were included (n = 461). Patients who withdrew their informed consent (n = 26) or for whom the uNAG measurement was not available (n = 45) were excluded, leaving 390 participants. The study conformed to the Declaration of Helsinki and was approved by the Institutional Review Board of the Second Affiliated Hospital of Zhejiang University. Written informed consent was provided by all patients.

### 2.2 Definition of HF and MR

In accordance with the ESC [22] and Chinese guidelines [23], a diagnosis of HF was based on the description of symptoms (chest tightness, dyspnea, exercise intolerance), physical examination (pulmonary rales or peripheral edema), laboratory measurements (B-type natriuretic peptide (BNP) >35 pg/mL or N-terminal pro-B-type natriuretic peptide (NT-proBNP) >125 pg/mL), chest X-rays and echocardiography. HF with reduced ejection fraction (HFrEF) was defined as an ejection fraction <40%, whereas HF with preserved ejection fraction (HFpEF) was defined as an ejection fraction ≥50% with at least one of the following: LA enlargement and/or left ventricular (LV) hypertrophy and/or E/e’ ≥13 (E/e’ refers to the ratio between the early diastolic velocity of mitral inflow and that of the mitral annulus). HF with mid-range ejection fraction (HFmrEF) was defined as an ejection fraction between 40–49% with at least one of the following: LA enlargement and/or LV hypertrophy and/or E/e’ ≥13.

MR was assessed quantitatively using the proximal isovelocity surface area (PISA) to calculate mitral regurgitation volume (RVol) and the effective regurgitation orifice area (EROA). The severity of MR was classified as grade 0 for no regurgitation, grade 1 for mild regurgitation (EROA <0.2 ${\mathrm{cm}}^{2}$ and/or RVol <30 mL), grade 2 for moderate regurgitation (0.3 ${\mathrm{cm}}^{2}$ > EROA ≥ 0.2 ${\mathrm{cm}}^{2}$ and/or 45 mL > RVol ≥ 30 mL), grade 3 for moderate to severe regurgitation (0.4 ${\mathrm{cm}}^{2}$ > EROA ≥ 0.3 ${\mathrm{cm}}^{2}$ and/or 60 mL > RVol ≥ 45 mL), and grade 4 for severe regurgitation (EROA ≥0.4 ${\mathrm{cm}}^{2}$ and/or RVol ≥60 mL) [24].

### 2.3 Data Collection

Baseline clinical data included the patient characteristics of age, gender, body-mass index (BMI), systolic blood pressure (SBP), diastolic blood pressure (DBP), heart rate, New York Heart Association (NYHA) functional class, comorbidities (diabetes, hypertension, atrial fibrillation, coronary artery disease (CAD) and chronic kidney disease (CKD, adjudicated according to medical records or estimated glomerular filtration rate (eGFR) <60 mL/min/1.73 $m^{2}$)), laboratory results, echocardiography parameters, MR grade, intravenous treatment during hospitalization, and oral medications at discharge.

### 2.4 Sample Collection

Venous blood and spot urine samples were obtained in the morning within 24 h of admission and immediately sent to the hospital’s central laboratory for measurement of routine clinical parameters. These included hemoglobin (Hb), NT-proBNP, C-reactive protein (CRP), serum sodium, serum creatinine (Scr), blood urea nitrogen (BUN), urinary NAG and microalbumin. Urinary NAG was measured with the NAG kit (MPT) as per the manufacturer’s instructions (Beijing Leadman Biochemistry Technology Co. Ltd. Beijing, China) on a Beckman Coulter instrument AU5800 (Beckman Coulter, Brea, CA, USA). Urinary microalbumin was measured using scatter turbidimetry on a special protein analyzer (BNII SYSTEM, Siemens, Munich, Germany). The Chronic Kidney Disease Epidemiology Collaboration (CKD-EPI) formula was used to calculate eGFR [25].

### 2.5 Echocardiography Measurement

A standard echocardiogram (Philips IE-33 color Doppler ultrasound imaging instrument, equipped with X-1 probe, S5 probe) was performed prior to discharge. In addition to the left ventricular ejection fraction (LVEF, based on the modified Simpson method), echocardiogram parameters included left atrium dimension (LAD), left ventricular end-diastolic volume (LVEDV), left ventricular internal diameter in diastolic phase (LVIDd), and left ventricular internal diameter in systolic phase (LVIDs). Echocardiography was performed and confirmed by experienced cardiac sonographers, with any discordant cases consulted further by a third sonographer.

### 2.6 Outcomes and Follow-Up

The primary endpoint was the composite of all-cause death (defined as death from any cause) or HF rehospitalization (defined as an inpatient admission with exacerbation of HF symptoms and requirement for treatment with intravenous diuretics or inotropic agent), while secondary outcomes included all-cause death and HF rehospitalization. All patients were followed up by outpatient visits or telephone contact at 1, 3, and 6 months after the date of index discharge, and every 6 months thereafter until death or the end of follow-up (2 years post-discharge). Patients lost to follow-up were censored at the time of last available contact.

### 2.7 Statistical Analysis

Normally distributed continuous variables were expressed as mean ± standard deviation (SD), while skewed distributed variables were presented as median and interquartile range (IQR). Categorical variables were expressed as numbers and percentages. Patients were classified into four groups according to the urinary NAG/urinary creatinine concentration ratio [26]. Differences between groups were evaluated using a one-way analysis of variance test, Kruskal-Wallis test, chi-squared test, or Fisher’s exact test where appropriate. Correlation analyses were examined by Spearman’s coefficient, since the distribution of uNAG values was non-normal. Associations between uNAG and endpoints were evaluated using the Kaplan-Meier survival method and compared using log-rank statistics. The receiver operating characteristic (ROC) curve was plotted and the area under the curve (AUC) was calculated to quantify the accuracy of the prediction. Univariable and multivariable Cox regression models were constructed to estimate the hazard ratio (HR) and 95% confidence interval (CI) of uNAG for the endpoints. uNAG was modeled as both categorical (quartiles) and continuous (per SD increase) variables. Traditional cardiovascular risk factors that can influence the prognosis of HF and MR based on previous literature were entered into the multivariable models. These included sex, age, CAD, hypertension, diabetes, CKD, NT-proBNP, LVEF, MR grade, intravenous use of diuretics, and urinary microalbumin. The linear relationship of uNAG with the incidence of study endpoints was evaluated using 3-knot restricted cubic splines. The concordance index (C-index) was used to evaluate whether NAG could provide additional prognostic value to the known prognostic factors. This is a generalization of the area under the ROC curve and is applicable to survival data. A C-index of 1 indicates perfect prediction accuracy, while a C-index of 0.5 indicates a random guess [27]. Subgroup analyses were performed according to age, gender, HF type, CAD/non-CAD, diabetes/non-diabetes, hypertension/non-hypertension, CKD/non-CKD, FMR/non-FMR and intravenous diuretics use/no intravenous diuretics use. Potential interactions were also tested. The R statistical software (version 4.2.2, R Foundation for Statistical Computing, Vienna, Austria) was used for all statistical analyses. A two-tailed *p*-value < 0.05 was considered statistically significant.

## 3. Results

### 3.1 Baseline Characteristics of HF Patients with MR

**Fig. 1.**
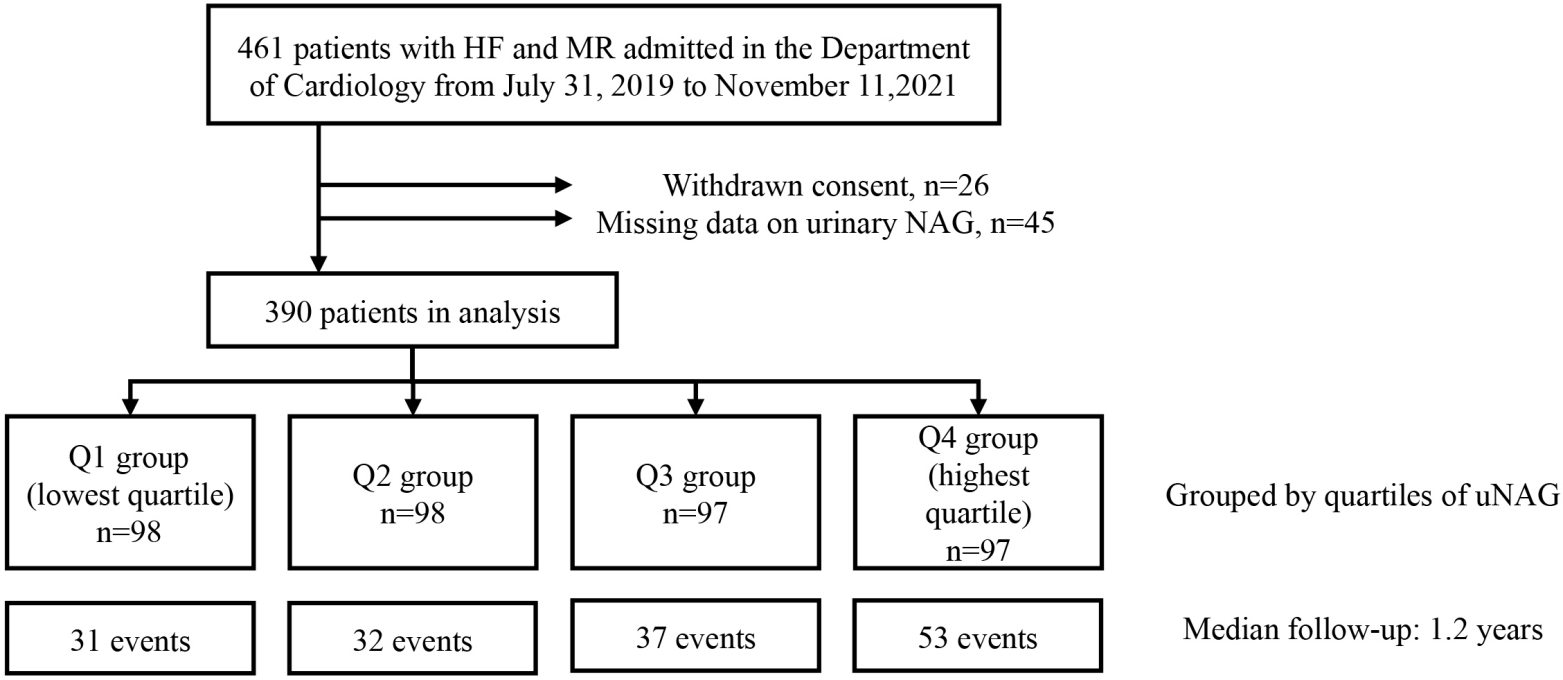
**Flow chart of subject selection**. Abbreviations: HF, heart failure; MR, mitral regurgitation; uNAG, urinary N-acetyl-β-d-glucosaminidase.

The study cohort was comprised of 390 patients with HF and MR (mean age 64 ± 14 years, 65.9% males) (Fig. 1). The median admission uNAG level was 8.08 U/g⋅Cr (IQR: 4.75–13.30). Patients were grouped according to the quartile of uNAG level. Baseline characteristics are presented in Table 1. The highest quartile uNAG group had a significantly higher prevalence of NYHA class III/IV and history of CKD. The highest quartile group was also associated with lower Hb and eGFR, and higher NT-proBNP, Scr, BUN, intravenous use of diuretics or vasoactive agents, and urinary microalbumin. **Supplementary Fig. 1** shows the boxplots of NAG concentrations across different MR grades. The differences between grades were statistically significant (*p* = 0.042).

**Table 1.** **Baseline characteristics for patients with HF and MR and classified according to quartiles of urinary NAG level**.

Variable		Overall	Q1 (<4.75)	Q2 (4.75 8.08)	Q3 (8.08 13.30)	Q4 (>13.30)	*p*-value
		N = 390	N = 98	N = 98	N = 97	N = 97	
Age, years		64 ± 14	62 ± 14	65 ± 11	65 ± 14	64 ± 15	0.363
Female, n (%)		134 (34.4)	37 (37.8)	35 (35.7)	27 (27.8)	35 (36.1)	0.468
BMI, kg/$m^{2}$		23.7 ± 4.0	24.1 ± 4.2	23.9 ± 3.2	23.7 ± 4.5	23.1 ± 4.2	0.305
SBP, mmHg		114.4 ± 17.8	112.8 ± 18.4	113.2 ± 16.9	116.3 ± 17.0	115.2 ± 18.8	0.466
DBP, mmHg		69.1 ± 13.7	69.4 ± 12.1	65.9 ± 12.4	70.6 ± 12.8	70.6 ± 16.6	0.050
Heart rate, bpm		79 ± 16	81 ± 15	77 ± 15	77 ± 17	81 ± 16	0.133
NYHA class							< **0.001**
	I, n (%)	35 (9.0)	11 (11.2)	13 (13.3)	7 (7.2)	4 (4.1)	
	II, n (%)	240 (61.5)	71 (72.4)	63 (64.3)	58 (59.8)	48 (49.5)	
	III, n (%)	98 (25.1)	13 (13.3)	20 (20.4)	26 (26.8)	39 (40.2)	
	IV, n (%)	17 (4.4)	3 (3.1)	2 (2.0)	6 (6.2)	6 (6.2)	
Comorbidity, n (%)							
	CAD	127 (32.6)	26 (26.5)	33 (33.7)	36 (37.1)	32 (33.0)	0.457
	Diabetes	98 (25.1)	23 (23.5)	24 (24.5)	20 (20.6)	31 (32.0)	0.306
	Hypertension	173 (44.4)	35 (35.7)	42 (42.9)	47 (48.5)	49 (50.5)	0.157
	Atrial fibrillation	140 (35.9)	34 (34.7)	34 (34.7)	38 (39.2)	34 (35.1)	0.895
	CKD	108 (27.7)	10 (10.2)	23 (23.5)	31 (32.0)	44 (45.4)	< **0.001**
Intravenous treatment, n (%)							
	Inotropic agent	110 (28.2)	19 (19.4)	24 (24.5)	24 (24.7)	43 (44.3)	< **0.001**
	Diuretics	219 (56.2)	42 (42.9)	53 (54.1)	55 (56.7)	69 (71.1)	**0.001**
	Vasodilator	26 (6.7)	0 (0.0)	3 (3.1)	9 (9.3)	14 (14.4)	< **0.001**
	Vasopressor	19 (4.9)	1 (1.0)	7 (7.1)	4 (4.1)	7 (7.2)	0.139
Prescriptions at discharge, n (%)							
	ACEI	13 (3.3)	4 (4.1)	4 (4.1)	5 (5.2)	0 (0.0)	0.196
	ARB	21 (5.4)	5 (5.1)	7 (7.1)	2 (2.1)	7 (7.2)	0.341
	ARNI	274 (70.3)	74 (75.5)	73 (74.5)	68 (70.1)	59 (60.8)	0.099
	Beta-blockers	285 (73.1)	79 (80.6)	71 (72.4)	73 (75.3)	62 (63.9)	0.065
	MRA	275 (70.5)	73 (74.5)	71 (72.4)	68 (70.1)	63 (64.9)	0.498
	Diuretics	306 (78.5)	72 (73.5)	77 (78.6)	76 (78.4)	81 (83.5)	0.406
Laboratory data at admission							
	Hb, mg/dL	128.7 ± 27.2	138.4 ± 21.1	128.3 ± 23.1	126.9 ± 31.6	120.9 ± 29.2	< **0.001**
	NT-proBNP, pg/mL	1404.0 (657.5, 3707.8)	850.0 (436.8, 2308.5)	1058.0 (528.0, 2189.0)	1518.0 (803.0, 4324.0)	3754.0 (1569.0, 8704.0)	< **0.001**
	CRP, mg/dL	5.9 (5.0, 15.3)	5.0 (5.0, 8.3)	5.0 (5.0, 12.0)	7.5 (5.0, 16.1)	9.8 (5.0, 28.8)	**0.006**
	Serum sodium, mmol/L	139.7 ± 3.6	139.8 ± 3.1	140.2 ± 3.5	140.1 ± 3.1	138.6 ± 4.4	**0.007**
	Scr, μmol/L	84.0 (69.0, 113.0)	75.0 (66.0, 86.6)	81.0 (66.3, 105.8)	97.0 (74.0, 118.0)	98.0 (78.0, 135.2)	< **0.001**
	BUN, mmol/L	7.2 (5.6, 9.9)	6.7 (5.0, 7.7)	6.7 (5.5, 8.5)	7.9 (6.1, 11.4)	9.1 (6.5, 13.5)	< **0.001**
	eGFR, mL/min·1.73 $m^{2}$	82.3 ± 35.1	97.0 ± 29.5	88.0 ± 35.8	74.7 ± 34.6	69.4 ± 33.8	< **0.001**
	Urinary microalbumin, mg/g·Cr	23.5 (12.4, 71.0)	12.4 (7.6, 23.9)	21.4 (12.4, 47.1)	27.0 (16.2, 91.7)	78.8 (25.0, 246.9)	< **0.001**
Echocardiography parameter							
	LVEF, %	31.9 (25.6, 41.9)	32.6 (26.1, 42.3)	34.1 (28.4, 42.5)	31.9 (25.4, 41.7)	29.3 (23.9, 41.8)	0.083
	LAD, cm	4.4 ± 0.8	4.3 ± 0.7	4.4 ± 0.8	4.5 ± 0.8	4.4 ± 0.7	0.241
	LVEDV, mL	168.0 ± 71.4	153.5 ± 51.9	167.7 ± 79.8	177.6 ± 78.7	172.4 ± 70.5	0.177
	LVIDd, cm	6.1 ± 1.1	6.0 ± 0.9	6.1 ± 1.1	6.3 ±1.2	6.1 ± 1.2	0.261
	LVIDs, cm	5.1 ± 1.3	5.0 ± 1.1	5.0 ± 1.3	5.3 ± 1.3	5.2 ± 1.4	0.279
MR grade							0.136
	1, n (%)	254 (65.1)	64 (65.3)	73 (74.5)	63 (64.9)	54 (55.7)	
	2, n (%)	86 (22.1)	22 (22.4)	16 (16.3)	24 (24.7)	24 (24.7)	
	3, n (%)	26 (6.7)	8 (8.2)	5 (5.1)	6 (6.2)	7 (7.2)	
	4, n (%)	24 (6.2)	4 (4.1)	4 (4.1)	4 (4.1)	12 (12.4)	

Values are expressed as mean ± standard deviation, median (interquartile range) or number (percentages). Bold font indicates statistical significance. Abbreviations: NAG, N-acetyl-β-d-glucosaminidase; BMI, body-mass index; SBP, systolic blood pressure; DBP, diastolic blood pressure; NYHA, New York Heart Association; CAD, coronary artery disease; CKD, chronic kidney disease; ACEI/ARB/ARNI, angiotensin converting enzyme inhibitor/angiotensin II receptor blocker/angiotensin receptor-neprilysin inhibitor; MRA, mineralocorticoid receptor antagonists; Hb, hemoglobin; NT-proBNP, N-terminal pro-B-type natriuretic peptide; CRP, C-reactive protein; Scr, serum creatinine; BUN, blood urea nitrogen; eGFR, estimated glomerular filtration rate; LVEF, left ventricular ejection fraction; LAD, left atrium dimension; LVEDV, left ventricular end-diastolic volume; LVIDd, left ventricular internal diameter in diastolic phase; LVIDs, left ventricular internal diameter in systolic phase; MR, mitral regurgitation; HF, heart failure.

### 3.2 Correlations between Urinary NAG levels and Clinical Variables

The results of correlation analyses between uNAG and other clinical variables are presented in Table 2. uNAG levels showed a significant positive correlation with NT-proBNP, CRP and urinary microalbumin. Significant negative correlations were found between uNAG levels and eGFR, Hb, LVEF, and serum sodium.

**Table 2.** **Correlation analyses of admission urinary NAG levels with clinical variables**.

Variables	Urinary NAG	
	r	*p-*value
Age, years	0.077	0.128
Body-mass index, kg/$m^{2}$	–0.104	**0.041**
Systolic blood pressure, mmHg	0.046	0.366
Diastolic blood pressure, mmHg	0.034	0.505
Heart rate, bpm	0.007	0.892
Hemoglobin, mg/dL	–0.198	< **0.001**
NT-proBNP, pg/mL	0.417	< **0.001**
C-reactive protein, mg/dL	0.215	< **0.001**
Serum sodium, mmol/L	–0.162	**0.001**
Serum creatinine, μmol/L	0.336	< **0.001**
eGFR, mL/min·1.73 $m^{2}$	–0.338	< **0.001**
Urinary microalbumin, mg/g·Cr	0.496	< **0.001**
LVEF, %	–0.116	**0.022**
LAD, cm	0.111	**0.028**
LVEDV, mL	0.106	0.061
LVIDd, cm	0.064	0.206
LVIDs, cm	0.088	0.081

Bold font indicates statistical significance. Abbreviations: NT-proBNP, N-terminal pro-B-type natriuretic peptide; eGFR, estimated glomerular filtration rate; LVEF, left ventricular ejection fraction; LAD, left atrium dimension; LVEDV, left ventricular end-diastolic volume; LVIDd, left ventricular internal diameter in diastolic phase; LVIDs, left ventricular internal diameter in systolic phase; NAG, N-acetyl-β-d-glucosaminidase.

### 3.3 Urinary NAG Levels and Clinical Outcomes

**Fig. 2.**
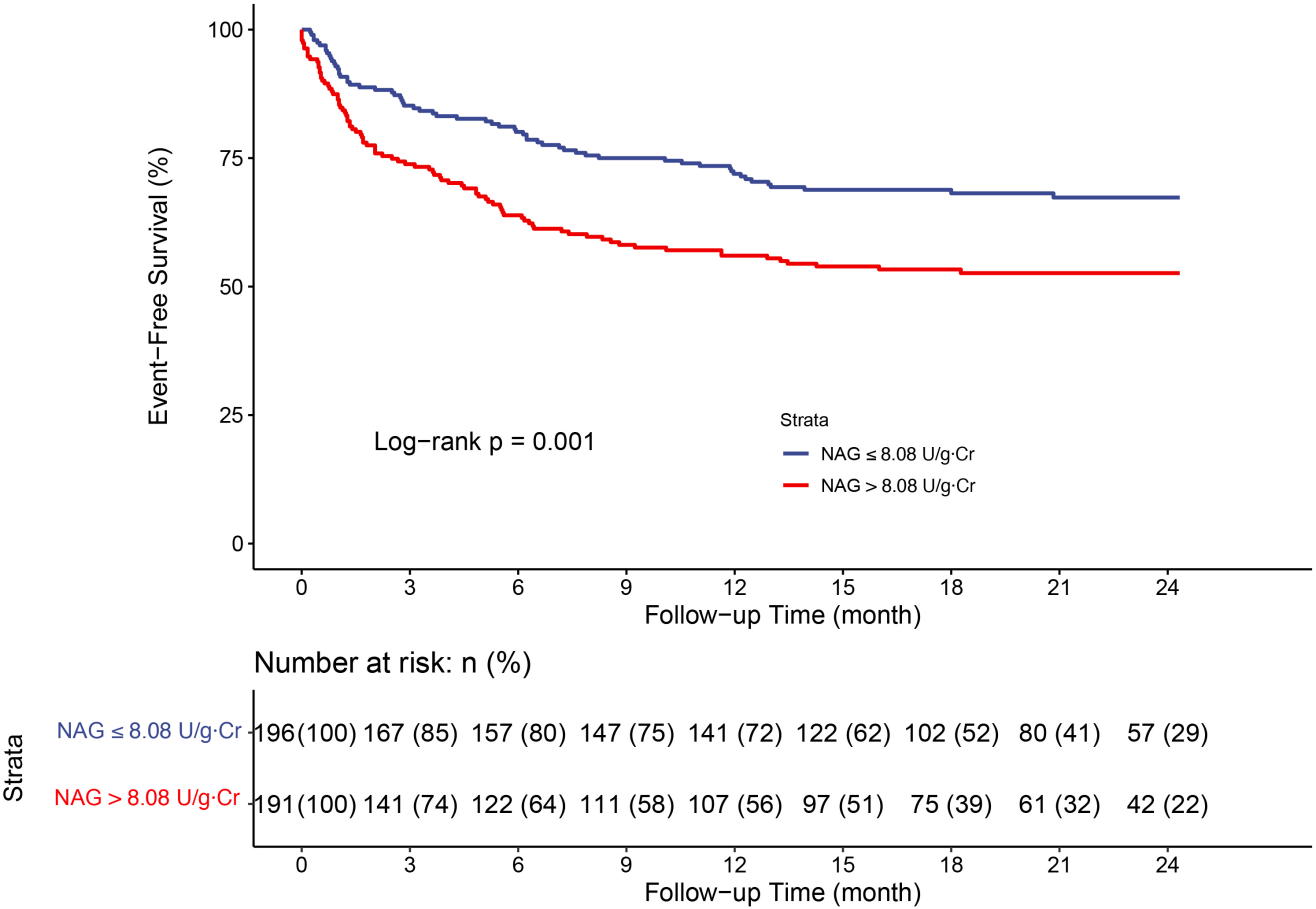
**Kaplan–Meier analysis for all-cause death or HF rehospitalization stratified by urinary NAG median**. Abbreviations: NAG, N-acetyl-β-d-glucosaminidase; HF, heart failure.

During the median follow-up of 1.2 years (IQR: 0.4–2.0 years), 3 of the 390 (0.8%) patients were lost to follow-up and 153 (35.5%) experienced a primary endpoint event (all causes of death (n = 52, 13.3%), HF rehospitalization (n = 126, 32.3%)). Kaplan–Meier analysis revealed that a higher uNAG level (≥8.08 U/g⋅Cr, which was the median level) was associated with significantly worse clinical outcomes (Fig. 2). The ROC curve had an AUC of 0.614 (95% CI, 0.553 to 0.671) (**Supplementary Fig. 2**).

**Fig. 3.**
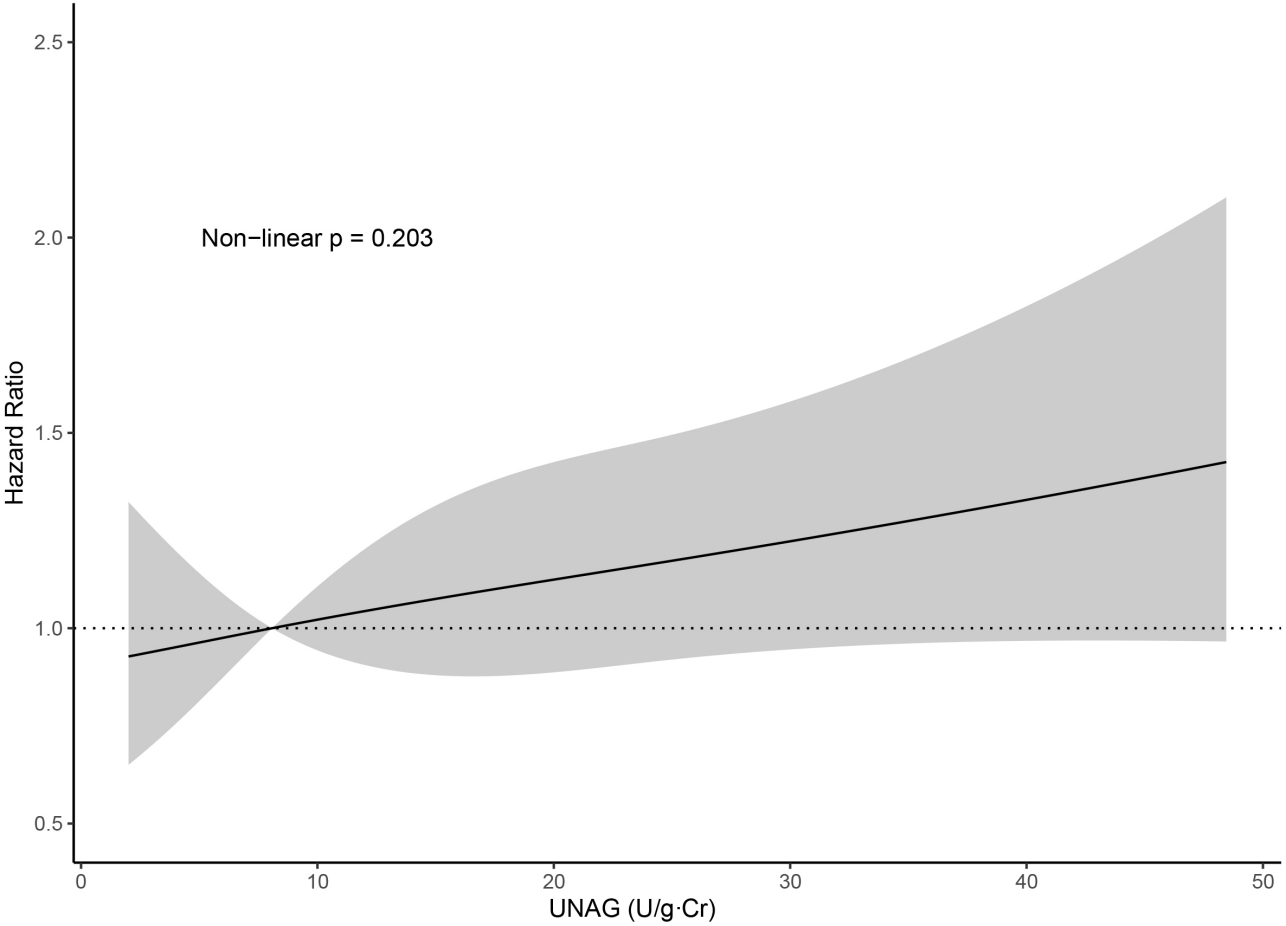
**Association between uNAG and all-cause death or HF rehospitalization, presented as the hazard ratio (solid line) and 95% confidence intervals (shaded area) and adjusted for sex, age, hypertension, diabetes, CAD, CKD, NT-proBNP, LVEF, MR grade, intravenous use of diuretics, and urinary microalbumin**. Abbreviations: uNAG, urinary N-acetyl-β-d-glucosaminidase; CAD, coronary artery disease; CKD, chronic kidney disease; NT-proBNP, N-terminal pro-B-type natriuretic peptide; LVEF, left ventricular ejection fraction; MR, mitral regurgitation; HF, heart failure.

In univariate analysis, higher uNAG level was associated with significantly increased risks for all-cause mortality, HF rehospitalization, and the composite of all-cause death or HF rehospitalization (**Supplementary Table 1**). Multivariable Cox analysis adjusted for sex, age, CAD, hypertension, diabetes, CKD, NT-proBNP, LVEF, MR grade, intravenous use of diuretics, and urinary microalbumin was performed. Each SD (13.80 U/g⋅Cr) of higher uNAG level was associated with a 17% higher risk of death or HF rehospitalization (95% CI, 2–33%, *p* = 0.022), and a 19% higher risk for HF rehospitalization (95% CI, 2–39%, *p* = 0.027). After adjusting for covariates, each increasing quartile of uNAG was no longer significantly associated with elevated hazard ratios for any adverse outcomes (Table 3, **Supplementary Tables 2,3**). Assessment of restricted cubic splines also supports a linear relationship between uNAG levels and the primary outcome (Fig. 3, *p* non-linear = 0.203). The corresponding C-index was also calculated in order to test the incremental prognostic value of uNAG. The addition of uNAG to a Cox regression model without uNAG yielded a small increase in the C-index value, from 0.7156 (95% CI, 0.6952–0.7360) to 0.7177 (95% CI, 0.6973–0.7318).

**Table 3.** **Cox proportional hazards model for the composite of all-cause mortality or HF rehospitalization**.

	Urinary NAG Quantiles				Continuous
	Q1 <4.75	Q2 4.75∼8.08	Q3 8.08∼13.30	Q4 >13.30	Per SD (13.80) greater
Events/N at risk	31/98	32/98	37/97	53/97	153/390
Unadjusted HR (95% CI)	1.00 (Ref.)	1.02 (0.63–1.68)	1.30 (0.81, 2.10)	2.19 (1.40–3.41)	1.31 (1.18–1.45)
Adjusted HR (95% CI) *	1.00 (Ref.)	0.96 (0.58–1.58)	0.98 (0.60, 1.61)	1.36 (0.83–2.21)	1.17 (1.02–1.33)

* Adjusted for sex, age, CAD, diabetes, hypertension, CKD, NT-proBNP, LVEF, MR grade, in-hospital use of intravenous diuretics and urinary microalbumin. Abbreviations: NAG, N-acetyl-β-d-glucosaminidase; CAD, coronary artery disease; CKD, chronic kidney disease; NT-proBNP, N-terminal pro-B-type natriuretic peptide; LVEF, left ventricular ejection fraction; MR, mitral regurgitation; HF, heart failure; HR, hazard ratio.

### 3.4 Subgroup Analysis

**Fig. 4.**
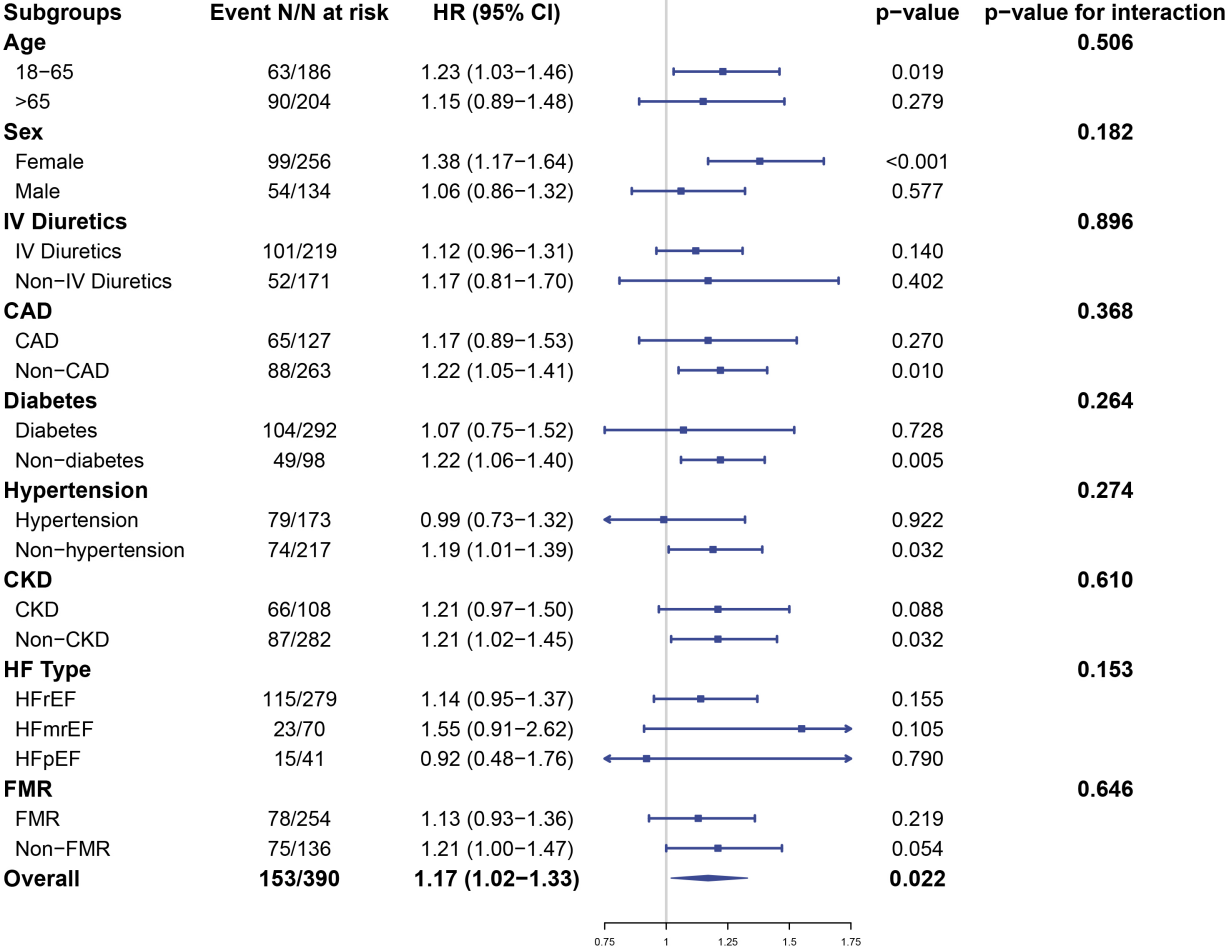
**Subgroup analysis showing the hazard ratio for urinary NAG (per SD: 13.80 U/g⋅Cr) for all-cause death and HF rehospitalization**. The analysis was adjusted for sex, age, hypertension, diabetes, CAD, CKD, NT-proBNP, LVEF, MR grade, intravenous use of diuretics, urinary microalbumin. Abbreviations: NAG, N-acetyl-β-d-glucosaminidase; CAD, coronary artery disease; CKD, chronic kidney disease; NT-proBNP, N-terminal pro-B-type natriuretic peptide; LVEF, left ventricular ejection fraction; MR, mitral regurgitation; HF, heart failure; HFrEF/HFpEF/HFmrEF, HF with reduced/preserved/mid range ejection fraction; FMR, functional mitral regurgitation.

Subgroup analyses were performed to determine whether uNAG levels had similar prognostic value in different populations. Except for the stratification variable, all analyses were adjusted for sex, age, CAD, hypertension, diabetes, CKD, NT-proBNP, LVEF, MR grade, intravenous use of diuretics and urinary microalbumin. As shown in Fig. 4, the association between uNAG and the composite endpoint was significant only in younger patients, female patients, and in patients without CAD, diabetes, hypertension, or CKD (all *p* < 0.05). No significant interactions were found between uNAG and the stratification factors (all *p* ≥ 0.05).

## 4. Discussion

The first major finding of this study was that uNAG levels correlated with the severity of HF. Second, the uNAG level in patients with HF and MR was independently associated with the composite of all-cause mortality or HF rehospitalization, with this association being almost linear. Third, subgroup analysis suggested the uNAG level at admission also had similar prognostic significance in younger patients, and in patients without comorbidities. To our knowledge, this study is the first to evaluate the association between uNAG as a tubular biomarker and adverse outcomes in patients with HF and MR. However, further studies are needed to evaluate the therapeutic implications of this finding.

### 4.1 Mitral Regurgitation and Renal Dysfunction

Renal dysfunction has long been considered one of the factors for poor prognosis in MR patients. An observational study conducted in 5213 patients who underwent MitraClip showed that preprocedural renal disease was common (77% with creatinine clearance <60 mL/min) and was associated with poor outcomes, with a 1-year mortality rate of almost one-third in stage 4/5 renal disease, and one-fifth in stage 3 renal disease [28]. Among patients undergoing MV surgery, those on dialysis had consistently lower survival rates compared to those not on dialysis (59.2% vs. 89.5% at 1-year, 28.9% vs. 78.4% at 5 years, and 19.6% vs. 63.9% at 10 years follow-up, respectively) [29]. However, Kainuma *et al*. [30] reported that MV repair yielded improvements in LV function and hemodynamics regardless of the preoperative renal function status, and that patients with ESRD had lower mortality and HF readmission rates than those with CKD. On the other hand, in patients with pre-existing renal insufficiency, successful MitraClip implantation led to improved eGFR in patients with increased forward stroke volume (FSV) [31]. Renal hemodynamic improvement brought about by reduced regurgitation volume and increased FSV through increased perfusion (via increased cardiac output) and decreased congestion (via decreased preload and decreased venous pressure) may account for the improved renal function. Recent studies found a 16–20% incidence of acute kidney injury after percutaneous MV repair, despite claims of “zero-contrast” [32, 33]. These findings imply a sophisticated cardiorenal pathophysiology.

### 4.2 N-Acetyl-β-d-Glucosaminidase and Cardiorenal Disease

There has been some research into the prognostic value of uNAG for worsening renal failure and adverse cardiovascular outcomes. Brankovic *et al*. [34] showed that an increase in the slope of uNAG levels was associated with a higher risk of composite endpoint in 263 chronic HF patients, with the association being stronger than that of plasma creatinine. Damman *et al*. [35] found that higher baseline uNAG was the strongest predictor of worse clinical outcome compared to other tubular markers. In a 10-year follow-up of 149 patients with chronic HF, Strack *et al*. [20] used multivariable Cox analysis to show that uNAG was a significant and independent predictor of all-cause mortality, but was no longer significant when combined with NT-proBNP. Moreover, longer follow-up could result in more bias. For example, it is not known whether the correlation between NT-proBNP and NAG changes over time, and whether or not a patient’s disease state changes drastically and is restored to the original state in the interim. In a large cohort of 2466 adults with CKD (eGFR of 20–70 mL/min per 1.73 $m^{2}$), Park *et al*. [36] showed after multivariable adjustment that uNAG/uCr was associated with mortality as a continuous variable, but not as quintiles. In contrast, Ahmad *et al*. [37] showed that an increase in any tubular injury biomarker (including uNAG) was not associated with worsening renal failure, but paradoxically to improved survival. However, their study involved patients with acute rather than chronic HF, and these were treated with aggressive diuresis such that effective decongestion associated with favourable outcomes may have been achieved [37]. It should be noticed that results across these studies should not be directly compared because of difference in study designs, treatment approaches and statistical analyses.

### 4.3 N-Acetyl-β-d-Glucosaminidase and Mitral Regurgitation

To our knowledge, this is the first study to investigate the relationship between tubular dysfunction or uNAG and MR. Patients with higher uNAG were found to have a lower baseline hemoglobin level. These patients may be more susceptible to renal tubular injury because of their limited capacity to endure chronic hypoxia. Moreover, there was a trend for higher rates of intravenous diuretic use in the highest uNAG quartile group, indicating the existence of a subclinical venous congestion state and elevated central venous pressure (CVP). Increased CVP has been associated with impaired renal function in patients with advanced HF [38, 39]. Several studies have highlighted the importance of adequate fluid removal and meticulous monitoring of volume status in MR patients. Preload/afterload-reducing medications such as diuretics, nitrates, hydralazines, or ultrafiltration are helpful in reducing the severity of MR [40, 41]. Among patients who underwent a restrictive mitral annuloplasty, those on hemodialysis showed favorable late outcomes compared to those not on hemodialysis [30]. Another study showed that more diuretic use was associated with worse renal function (higher creatinine) and worse prognosis [42]. However, patients with acute decompensated HF treated with diuretics may show increased serum creatinine, but this may simply indicate effective tissue de-edema therapy, which is associated with better outcomes [43]. These findings suggest that the context in which renal dysfunction develops, rather than simply its presence, is the primary determinant of adverse outcomes. Intrarenal physiological changes may be clinically benign and therefore followed with a good prognosis. Further studies are required to elucidate the involved pathophysiology and to further our understanding of cardiorenal syndrome, including the right heart-kidney interaction [44].

### 4.4 Study Implications

A very recent study has suggested that eGFR declines prior to hospitalization for HF, thus highlighting the preadmission period as high-risk and an important opportunity to initiate or up-titrate medications [45]. Monitoring of kidney functions such as the eGFR trajectory may identify patients who are at high risk of clinical deterioration. Serum creatinine (Scr) and creatinine clearance (Ccr) are used to reflect renal injury. However, frequent measurements of Scr can harm patients, while muscle mass, diet and some evidence-based drugs may influence creatinine levels. Hence, more reliable and non-invasive biomarkers are needed. Considering the relative stability and noninvasive testing of uNAG, along with the present study, early recognition of at-risk patients may be achieved by monitoring the trajectory of urinary tubular injury markers.

An meaningful finding of our study was that uNAG was associated with the single endpoint of HF rehospitalization. Advanced HF was characterized by worsening symptoms, recurrent hospitalizations, and greater lengths of hospital stay, incurring significant financial burdens to the patient and the healthcare system. Heidenreich *et al*. [46] estimated that hospitalization for HF would account for 80% of the cost for care of HF patients. In China, the inpatient cost among urban HF patients accounted for 66% of their total cost [47]. The economic implications of rehospitalizations are self-evident, and substantial savings in healthcare system will be achieved if we can reduce HF admission rate. Our observation that uNAG played a role in predicting rehospitalizations emphasized the possibility of uNAG being used as a prognostic marker. Urine-based biomarker monitoring in clinical practice is repeatable and cost-effective. Given the large number of patients with HF and MR and the ease and low cost of urine sample collection and analysis, monitoring uNAG for early identification and outpatient intervention of high-risk patients should lead to improved outcomes as well as reduced health expenditure.

So far, the effects of percutaneous therapy on the MR population have given opposite, and yet complementary results [48, 49]. Following in-depth analysis and comparison, it was concluded that appropriately selected patients (disproportionate severe MR with cardiac function) may benefit from percutaneous therapy. In light of our finding that admission uNAG level was an independent prognostic factor for patients with MR, this raises the question of whether baseline clinical test indicators could improve candidate selection for intervention. Unfortunately, research in this area is still sparse and our study was mainly hypothesis generating in nature. More studies are needed to confirm our conclusions and to elucidate the cause-effect relationship for higher uNAG levels being associated with poorer prognosis, as well as whether tubular dysfunction could be a potential target for MR therapeutics.

In summary, this study has advanced our understanding of cardiorenal interactions in MR, its impact on patient manifestations during hospitalization, and on the clinical outcomes after discharge. Confirmation of the link between tubular dysfunction (as indicated by urinary NAG levels) and MR will give physicians a cheap and non-invasive biomarker to facilitate decision making and reduce healthcare costs.

### 4.5 Limitations

There are several limitations to this study. First, extrapolation of the results are limited by the single-center and observational nature of the study. The relatively small sample size might also have introduced selection bias. Second, the AUC value of NAG was relatively low. We speculate that the preliminary nature of this work may limit the power to make robust conclusions but lays the foundation for future studies. We believed that studies with a larger sample size and longer follow-up may achieve superior predictive accuracy. Third, the improvement in the C-index was small and we therefore had insufficient power to demonstrate the additional prognostic value of NAG levels in this cohort, particularly in comparison to NT-pro BNP. Given the multifactorial nature and complex pathophysiology of MR, it may be that the prognosis of patients with MR is also dependent upon other clinical characteristics (e.g., the duration of illness, baseline cardiac function) rather than solely renal function, including tubular function. Our study was not designed to generate a prognostic model for use, but merely to explore the association between urinary NAG levels and the risk of adverse events in patients with MR. After adjustment of multiple confounders, NAG remained an independent predictor of HF rehospitalization and the composite endpoint of HF rehospitalization and death. Nonetheless, the predictive value of NAG for MR risk stratification purposes needs to be formally assessed and our findings need to be replicated in a different cohorts before these could be applied in clinical practice. Fourth, the relationship between uNAG and volume status could not be assessed because right heart catheterization was not performed during inpatient treatment. Furthermore, it was not known whether uNAG values were affected by the use of diuretics prior to hospitalization. Fifth, urinary NAG levels may fluctuate with disease progression and treatment application. A single measurement upon admission may therefore fail to track longitudinal changes and hence misrepresent its prognostic significance. Finally, a longer follow-up period should provide more accurate and complete information on the prognostic significance of uNAG.

## 5. Conclusions

We demonstrated that higher urinary NAG levels in patients with HF and MR can independently predict the risk of all-cause death or HF rehospitalization. These findings suggest that the uNAG level at admission may be a novel prognostic factor in patients with HF and MR.

## Data Availability

The datasets analyzed during the current study are available from the corresponding author on reasonable request.

## Abbreviations

MR, mitral regurgitation; HF, heart failure; uNAG, urinary N-acetyl-β-d-glucosaminidase; FMR, functional mitral regurgitation; LA, left atrial; ESRD, end-stage renal disease; MV, mitral valve; BNP, B type natriuretic peptide; NT-proBNP, N-terminal pro-B-type natriuretic peptide; E/e’, the ratio between the early diastolic velocity of mitral inflow and that of the mitral annulus; HFrEF/HFpEF/HFmrEF, HF with reduced/preserved/mid-range ejection fraction; LV, left ventricular; PISA, proximal isovelocity surface area; RVol, regurgitation volume; EROA, effective regurgitation orifice area; BMI, body-mass index; SBP, systolic blood pressure; DBP, diastolic blood pressure; NYHA, New York Heart Association functional class; CAD, coronary artery disease; CKD, chronic kidney disease; eGFR, estimated glomerular filtration rate; Hb, hemoglobin; CRP, C-reactive protein; Scr, serum creatinine; BUN, blood urea nitrogen; LVEF, left ventricular ejection fraction; LAD, left atrium dimension; LVEDV, left ventricular end-diastolic volume; LVIDd, left ventricular internal diameter in diastolic phase; LVIDs, left ventricular internal diameter in systolic phase; FSV, forward stroke volume; CVP, central venous pressure; SD, standard deviation; IQR, interquartile range; HR, hazard ratio; CI, confidence interval.

## Supplementary Material

Supplementary material associated with this article can be found, in the online version, at https://doi.org/10.31083/j.rcm2408219.
