# Supplementary material for: Prognostic Value of Urinary N-Acetyl-β-d-Glucosaminidase as a Marker of Tubular Damage in Patients with Heart Failure and Mitral Regurgitation
Source: Rev Cardiovasc Med. 2023 Jul 31;24(8):219. doi: 10.31083/j.rcm2408219 (PMC11266753; doi:10.31083/j.rcm2408219)
Supplement: Supplementary file 1 [file 2153-8174-24-8-219-s1.zip › 2153-8174-24-8-219-s1/Supplementary Tables.docx]

**Supplementary Table 1 Univariate and multivariate Cox regression model for all-cause death or HF rehospitalization**

|  | Univariable Model | |  | Multivariable Model (as quantiles) | |  | Multivariable Model (as continuous) | |
| --- | --- | --- | --- | --- | --- | --- | --- | --- |
| Characteristic | HR (95%CI) | P value |  | HR (95%CI) | *p*-value |  | HR (95%CI) | *p*-value |
| Age | 1.02 (1.00, 1.03) | **0.014** |  | 1.00 (0.98, 1.01) | 0.667 |  | 1.00 (0.98, 1.01) | 0.761 |
| Female | 1.03 (0.74, 1.44) | 0.860 |  | 1.20 (0.83, 1.73) | 0.337 |  | 1.18 (0.82, 1.70) | 0.373 |
| NYHA class |  |  |  |  |  |  |  |  |
| I | – | – |  |  |  |  |  |  |
| II | 1.49 (0.72, 3.08) | 0.284 |  |  |  |  |  |  |
| III | 3.02 (1.44, 6.36) | **0.004** |  |  |  |  |  |  |
| IV | 5.67 (2.31, 13.9) | **<0.001** |  |  |  |  |  |  |
| CAD | 1.69 (1.23, 2.33) | **0.001** |  | 1.37 (0.94, 1.99) | 0.104 |  | 1.35 (0.93, 1.97) | 0.113 |
| Diabetes | 1.61 (1.14, 2.25) | **0.006** |  | 1.21 (0.82, 1.78) | 0.348 |  | 1.23 (0.84, 1.81) | 0.295 |
| Hypertension | 1.43 (1.04, 1.97) | **0.026** |  | 1.17 (0.82, 1.65) | 0.389 |  | 1.19 (0.84, 1.69) | 0.325 |
| Atrial fibrillation | 0.88 (0.63, 1.23) | 0.465 |  |  |  |  |  |  |
| CKD | 2.57 (1.87, 3.54) | **<0.001** |  | 1.75 (1.21, 2.54) | **0.003** |  | 1.75 (1.21, 2.53) | **0.003** |
| IV inotropic agent | 1.87 (1.35, 2.60) | **<0.001** |  |  |  |  |  |  |
| IV Diuretics | 1.77 (1.27, 2.48) | **<0.001** |  | 1.49 (1.03, 2.15) | **0.033** |  | 1.47 (1.02, 2.12) | **0.040** |
| IV vasodilator | 2.33 (1.41, 3.87) | **0.001** |  |  |  |  |  |  |
| IV vasopressor | 1.96 (1.03, 3.71) | **0.040** |  |  |  |  |  |  |
| Diuretics | 1.50 (0.98, 2.31) | 0.062 |  |  |  |  |  |  |
| ACEI | 0.65 (0.24, 1.76) | 0.401 |  |  |  |  |  |  |
| ARB | 0.41 (0.15, 1.10) | 0.076 |  |  |  |  |  |  |
| ARNI | 0.65 (0.47, 0.90) | **0.010** |  |  |  |  |  |  |
| Beta-blockers | 0.58 (0.42, 0.81) | **0.002** |  |  |  |  |  |  |
| MRA | 0.89 (0.63, 1.26) | 0.525 |  |  |  |  |  |  |
| Hb, mg/dL | 0.98 (0.98, 0.99) | **<0.001** |  |  |  |  |  |  |
| NT-proBNP, pg/mL | 1.00 (1.00, 1.00) | **<0.001** |  | 1.00 (1.00, 1.00) | **<0.001** |  | 1.00 (1.00, 1.00) | **<0.001** |
| Serum sodium, mmol/L | 0.96 (0.91, 1.00) | 0.067 |  |  |  |  |  |  |
| Scr, mmol/L | 1.00 (1.00, 1.01) | **<0.001** |  |  |  |  |  |  |
| eGFR, mL/min·1.73m^2^ | 0.98 (0.98, 0.99) | **<0.001** |  |  |  |  |  |  |
| NAG grade |  |  |  |  |  |  |  |  |
| Q1 <4.75 | – | – |  | – | – |  |  |  |
| Q2 4.75~8.08 | 1.02 (0.63, 1.68) | 0.924 |  | 0.96 (0.58, 1.58) | 0.863 |  |  |  |
| Q3 8.08~13.30 | 1.30 (0.81, 2.10) | 0.281 |  | 0.98 (0.60, 1.61) | 0.932 |  |  |  |
| Q4 >13.30 | 2.19 (1.40, 3.41) | **<0.001** |  | 1.36 (0.83, 2.21) | 0.222 |  |  |  |
| NAG per SD, U/g·Cr | 1.31 (1.18, 1.45) | **<0.001** |  |  |  |  | 1.17 (1.02, 1.33) | **0.022** |
| Urinary microalbumin, mg/g·Cr | 1.00 (1.00, 1.00) | **0.021** |  | 1.00 (1.00, 1.00) | 0.127 |  | 1.00 (1.00, 1.00) | 0.185 |
| LVEF, % | 0.99 (0.98, 1.01) | 0.363 |  | 1.01 (1.00, 1.03) | 0.169 |  | 1.01 (1.00, 1.03) | 0.175 |
| MR grade |  |  |  |  |  |  |  |  |
| 1 | – | – |  | – | – |  | – | – |
| 2 | 2.13 (1.47, 3.07) | **<0.001** |  | 1.60 (1.08, 2.37) | **0.020** |  | 1.58 (1.07, 2.34) | **0.022** |
| 3 | 2.27 (1.28, 4.01) | **0.005** |  | 1.48 (0.80, 2.73) | 0.210 |  | 1.42 (0.77, 2.62) | 0.260 |
| 4 | 3.41 (1.99, 5.85) | **<0.001** |  | 2.28 (1.27, 4.10) | **0.006** |  | 2.41 (1.35, 4.31) | **0.003** |

Adjusted for sex, age, CAD, diabetes, hypertension, CKD, NT-proBNP, LVEF, MR grade, in-hospital use of intravenous diuretics and urinary microalbumin. Abbreviations: NAG, N-Acetyl-β-d-Glucosaminidase; NYHA, New York Heart Association; CAD, coronary artery disease; CKD, chronic kidney disease; IV, intravenous; ACEI/ARB/ARNI, angiotensin converting enzyme inhibitor/angiotensin II receptor blocker/angiotensin receptor-neprilysin inhibitor; MRA, mineralocorticoid receptor antagonists; Hb, Hemoglobin; NT-proBNP, N-terminal pro-B-type natriuretic peptide; Scr, serum creatinine; eGFR, estimated glomerular filtration rate; LVEF, left ventricular ejection fraction; MR, mitral regurgitation;

**Supplementary Table 2** Cox proportional hazards model for all-cause death

|  | Urinary NAG Quantiles | | | |  | Continuous |
| --- | --- | --- | --- | --- | --- | --- |
|  | Q1 <4.75 | Q2 4.75~8.08 | Q3 8.08~13.30 | Q4 >13.30 |  | Per SD (13.80) greater |
| Events/N at risk | 9/98 | 7/98 | 12/97 | 24/97 |  | 52/390 |
| Unadjusted HR (95%CI) | 1.00 (Ref.) | 0.77 (0.29-2.07) | 1.41 (0.59, 3.34) | 3.04 (1.41-6.55) |  | 1.31 (1.13-1.52) |
| Adjusted HR (95%CI) * | 1.00 (Ref.) | 0.72 (0.26-1.98) | 1.17 (0.48, 2.87) | 1.22 (0.50-2.99) |  | 1.09 (0.89-1.34) |

* Adjusted for sex, age, CAD, diabetes, hypertension, CKD, NT-proBNP, LVEF, MR grade, in-hospital use of intravenous diuretics and urinary microalbumin. Abbreviations: NAG, N-Acetyl-β-d-Glucosaminidase; CAD, coronary artery disease; CKD, chronic kidney disease; NT-proBNP, N-terminal pro-B-type natriuretic peptide; LVEF, left ventricular ejection fraction; MR, Mitral regurgitation.

**Supplementary Table 3** Cox proportional hazards model for heart failure rehospitalization

|  | Urinary NAG Quantiles | | | |  | Continuous |
| --- | --- | --- | --- | --- | --- | --- |
|  | Q1 <4.75 | Q2 4.75~8.08 | Q3 8.08~13.30 | Q4 >13.30 |  | Per SD (13.80) greater |
| Events/N at risk | 26/98 | 30/98 | 29/97 | 41/97 |  | 126/390 |
| Unadjusted HR (95%CI) | 1.00 (Ref.) | 1.16 (0.69-1.96) | 1.17 (0.69, 1.98) | 1.82 (1.11, 2.97) |  | 1.27 (1.12, 1.44) |
| Adjusted HR (95%CI) * | 1.00 (Ref.) | 1.15 (0.68-1.97) | 0.95 (0.55, 1.65) | 1.29 (0.74-2.23) |  | 1.19 (1.02-1.39) |

* Adjusted for sex, age, CAD, diabetes, hypertension, CKD, NT-proBNP, LVEF, MR grade, in-hospital use of intravenous diuretics and urinary microalbumin. Abbreviations: NAG, N-Acetyl-β-d-Glucosaminidase**;** CAD, coronary artery disease; CKD, chronic kidney disease; LVEF, left ventricular ejection fraction; NT-proBNP, N-terminal pro-B-type natriuretic peptide; MR, Mitral regurgitation.
