# Supplementary figures and images for: Prognostic Value of Urinary N-Acetyl-β-d-Glucosaminidase as a Marker of Tubular Damage in Patients with Heart Failure and Mitral Regurgitation
Source: Rev Cardiovasc Med. 2023 Jul 31;24(8):219. doi: 10.31083/j.rcm2408219 (PMC11266753; doi:10.31083/j.rcm2408219)

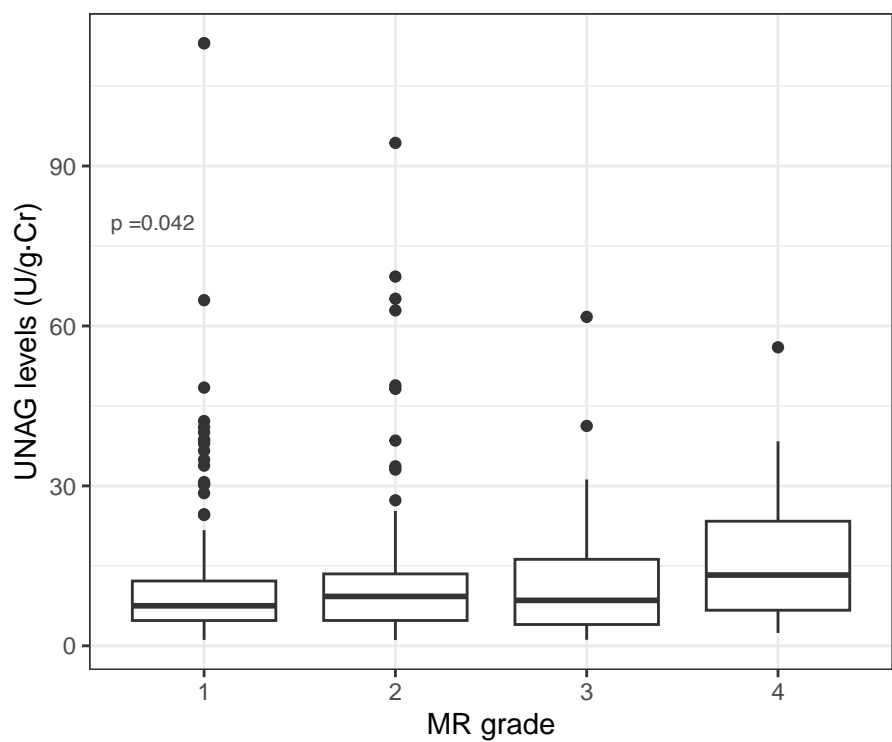

Supplement: Supplementary file 1 [file 2153-8174-24-8-219-s1.zip › 2153-8174-24-8-219-s1/Supplementary Fig.1_Boxplot.pdf]

## ROC Curve of UNAG

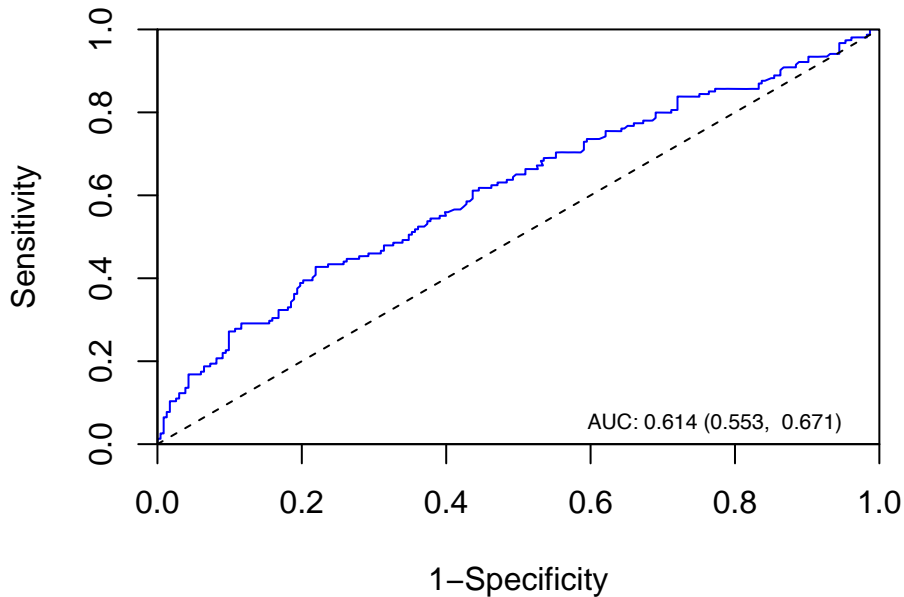

Supplement: Supplementary file 1 [file 2153-8174-24-8-219-s1.zip › 2153-8174-24-8-219-s1/Supplementary Fig.2_NAG_ROC.pdf]
